# MBD2 as a Potential Novel Biomarker for Identifying Severe Asthma With Different Endotypes

**DOI:** 10.3389/fmed.2021.693605

**Published:** 2021-10-05

**Authors:** Zhifeng Chen, Yu Yuan, Yi He, Binaya Wasti, Wentao Duan, Jingsi Jia, Danhong Li, Bing Xiao, Dongshan Zhang, Libing Ma, Jianmin Li, Yi Liu, Qingping Zeng, Xudong Xiang, Xiufeng Zhang, Shaokun Liu

**Affiliations:** ^1^Department of Respiratory Medicine, Research Unit of Respiratory Diseases, Hunan Centre for Evidence-Based Medicine, The Second Xiangya Hospital, Central South University, Changsha, China; ^2^Department of Emergency, The Second Xiangya Hospital, Central South University, Changsha, China; ^3^Department of Respiratory and Critical Care Medicine, The Affiliated Hospital of Guilin Medical University, Guilin, China; ^4^Department of Respiratory and Critical Care Medicine, Hunan Provincial People's Hospital, Changsha, China; ^5^Department of Respiratory Medicine, Zhuzhou City Central Hospital, Zhuzhou, China; ^6^Department of Respiratory and Critical Care Medicine, Longshan County People's Hospital, Longshan, China; ^7^Department of Respiratory Medicine, The Second Affiliated Hospital of Hainan Medical University, Haikou, China

**Keywords:** MBD2, severe asthma, T2, Th17, endotypes

## Abstract

**Background:** Studies have shown that methyl-CpG binding domain protein 2 (MBD2) expression is significantly elevated in a neutrophil-dominant severe asthma mouse model. It also regulates Th17 cell differentiation. The objective of this study was to investigate the relationship between serum MBD2 levels in patients with severe asthma with different endotypes.

**Methods:** Eligible adults with confirmed asthma (*n* = 63) underwent a clinical assessment, asthma control test and pulmonary function test and were classified as having mild, moderate or severe asthma. Severe asthma endotypes were defined according to the percentage of Th2 and Th17 cells in the peripheral blood and by the type of inflammation. The percentage of Th2 and Th17 cells in the peripheral blood was determined by flow cytometry. Serum MBD2, eosinophilic cationic protein and myeloperoxidase were measured by enzyme-linked immunosorbent assay. Correlations of MBD2 expression with clinical parameters were evaluated using Spearman's rank correlation analysis.

**Results:** Serum MBD2 levels were upregulated in patients with severe asthma compared to healthy controls and patients with mild to moderate asthma. MBD2 was also significantly increased in patients with Th17 severe asthma compared to patients with type 2 severe asthma. Furthermore, MBD2 was positively correlated with MPO and Th17 cells but negatively correlated with ECP and Th2 cells in patients with severe asthma.

**Conclusions:** These findings suggest that serum MBD2 may be a potential new biomarker for identifying severe asthma, Th17 severe asthma and the type of airway inflammation. However, these findings are still preliminary and need to be further investigated.

## Introduction

Asthma is a highly heterogeneous and prevalent chronic respiratory disease that affects ~300 million people worldwide (1). The inflammatory pathways of asthma involve multiple immune-driven mechanisms. T helper type 2 (Th2) cells and T helper type 17 (Th17) cells are subtypes of differentiated CD4^+^ T cells that play an important role in the immune response (2). Distinct asthma endotypes are used to describe asthma pathogenesis at the cellular and molecular levels (3). Currently, most studies classify asthma into type 2 (T2) and non-T2 endotypes (4, 5). The Th2-mediated pathway is thought to be the driving force for allergic asthma, and sputum eosinophil counts have been identified as biomarkers for this pathway, known as T2 asthma (6). However, studies have found that almost 50% of asthma cases show no infiltration of eosinophils (a non-T2 endotype) but are instead mainly infiltrated by neutrophils and insensitive to glucocorticoids. This pathway is mainly mediated by Th17 cells, known as Th17 asthma (or neutrophilic asthma) (7, 8). There are currently no markers for this endotype, and patients are more likely to develop severe asthma because of their insensitivity to glucocorticoids. International guidelines define “severe asthma” as asthma that required treatment at steps 4 or 5 of the Global Initiative for Asthma (GINA) in the previous year or systemic corticosteroids for ≥50% of the previous year to prevent it from becoming “uncontrolled,” or asthma that remains “uncontrolled” despite the use of this therapy (9). Therefore, the discovery of potential biomarkers to identify severe asthma endotypes is necessary, as this will improve disease control and optimize treatment outcomes.

DNA methylation is one of the epigenetic regulatory mechanisms of asthma, and the methyl-CpG binding domain (MBD) family of proteins play the role of “reader” and regulate of the epigenome in DNA methylation. One member of this family is MBD2 (10, 11). Zhong et al. found that MBD2 deleting leads to the inability to read methylation information, which disrupts the homeostasis of the T-bet/H 2.0-like homeobox axis and inhibits Th17 cell differentiation, thus having a protective effect on experimental autoimmune encephalomyelitis (12). Another study found that MBD2 was significantly elevated in mice with neutrophils-dominant asthma compared to mice with conventional asthma and the control group. Furthermore, MBD2 regulates Th17 cell differentiation and is positively correlated with Th17 cells (13). Eosinophilic cationic protein (ECP) is a marker of eosinophilic degranulation and activity, while myeloperoxidase (MPO) is a marker of neutrophil activation and adhesion (14, 15). Currently, biomarkers for T2 severe asthma include blood eosinophil count, fractional exhaled nitric oxide, serum ECP and serum periostin (16, 17). However, biomarkers for Th17 severe asthma have not been reported, and serum MBD2 levels in asthmatic patients have not been reported in the literature. Therefore, we hypothesized that MBD2 might be a potential biomarker for Th17 severe asthma.

In this study, the asthma endotype was determined based on the percentage of Th2 and Th17 cells in the peripheral blood of patients with severe asthma and by the type of inflammation. The correlation between serum MBD2 expression and clinical parameters was also analyzed. We confirmed that MBD2 expression was increased in patients with Th17 severe asthma and was associated with reduced lung function, suggesting that serum MBD2 contributes to the inflammatory process in patients with Th17 severe asthma.

## Materials and Methods

### Subjects

**Figure 1:**
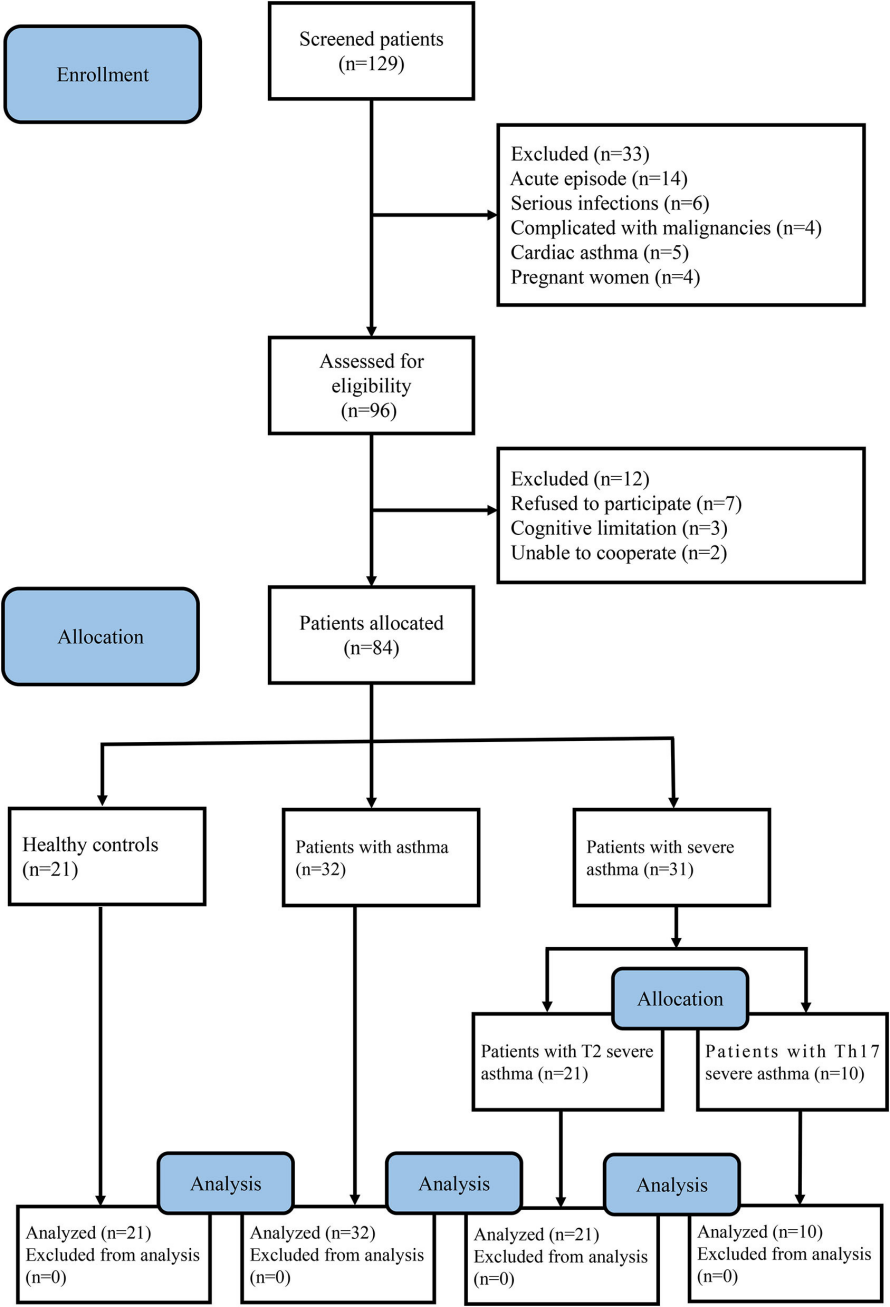
Flow diagram of the study.

We recruited all voluntary patients between April 10, 2020 and February 20, 2021. All voluntary patients had never smoked and ex-smokers with less than a 10 pack-year smoking history. Asthma control was assessed with an asthma control test (ACT) (18), scored on a scale of 0–25, where scores of 20–25 indicate well-controlled asthma and scores below 20 indicate that the patient felt symptomatic and that their asthma is poorly controlled. Asthma severity was evaluated according to ACT, pulmonary function test (PFT) and the therapeutic regimens of asthma patients, who were classified as having mild, moderate or severe asthma (19, 20). In order to distinguish them from severe asthma, mild and moderate asthma was classified as “asthma.” Severe asthma endotypes were defined according to the percentage of Th2 and Th17 cells in the peripheral blood and by the type of inflammation, which were divided into T2 severe asthma and Th17 severe asthma. The percentage of Th2 and Th17 cells in the peripheral blood was measured by flow cytometry. Adults with severe asthma (*n* = 31), asthma (*n* = 32) and healthy controls (HCs, *n* = 21) were enrolled at The Second Xiangya Hospital of Central South University (Hunan, China). The following inclusion criteria were used for patients with severe asthma: (a) asthma diagnosed according to GINA (21) with bronchodilation forced expiratory volume in 1 s (FEV_1_) change >200 ml and 12%; (b) had not received systemic corticosteroid treatment in the last 3 months; (c) FEV_1_%predicted <60% or peak expiratory flow (PEF) after initial bronchodilator percentage predicted <60% predicted; (d) frequent daily symptoms of asthma attacks, including wheezing, chest tightness, dyspnea, worsening cough, limited physical activity, and ACT <20; (e) the need for step 4 or 5 treatment regimens; and (f) at least 18 years of age. And the following patients were excluded: (a) acute episode, bronchogenic carcinoma, cardiac asthma, or allergic bronchopulmonary aspergillosis; (b) suffering from autoimmune disorders, hematological diseases or serious infections; (c) complicated with malignancies or solid tumors; and (d) pregnant or lactating women. The following inclusion criteria were used for patients with asthma: (a) asthma diagnosed according to GINA (21) with bronchodilation FEV_1_ change >200 ml and 12%; (b) had not received systemic corticosteroid treatment in the last 3 months; (c) FEV_1_%predicted >60% or PEF after initial bronchodilator percentage predicted >60% predicted; (d) asthma symptoms (including wheezing, dyspnea, chest tightness, or coughing) occur occasionally, may interfere with activity and sleep and ACT >20; (e) no need for step 4 or 5 treatment regimens; and (f) at least 18 years of age. And the following patients were excluded: (a) acute episode, bronchogenic carcinoma, cardiac asthma, or allergic bronchopulmonary aspergillosis; (b) suffering from autoimmune disorders, hematological diseases, neoplastic diseases or serious infections; (c) complicated with malignancies or solid tumors; and (d) pregnant or lactating women. HCs had no history of chronic respiratory disease and were not atopic. Atopy is defined as an individual and/or familial tendency, usually in childhood or adolescence, to become sensitized and produce IgE antibodies in response to exposure to allergens, usually proteins (22). In this study, atopy was excluded by no history of allergic dermatitis, urticaria, allergic rhinitis or other allergy-related conditions, and a negative skin-prick test. A detailed description of the flow diagram for recruiting voluntary patients can be found in Figure 1.

### Data Collection

After the collection of written informed consent, participants' age and gender were documented. Height and weight were measured, and body mass index (BMI) was calculated. Meanwhile, laboratory tests (e.g., immuno globulin E [IgE], blood ensinophils, blood neutrophils) and PFT results were recorded, which included FEV_1_ and forced vital capacity (FVC), and the values of FEV_1_/FVC as well as FEV_1_%predicted were calculated.

### Blood Sample Collection, Isolation of Peripheral Blood Mononuclear Cells, Culture Condition, and Reagents

Whole blood (5 ml) from each subject was collected in EDTA tubes, and peripheral blood mononuclear cells (PBMCs) were isolated by density centrifugation with Ficoll-Paque (23, 24). Briefly, whole blood was collected and overlayed on the liquid surface of the human peripheral blood lymphocyte separation solution (TBD sciences, Tianjin, China), followed by centrifugation (600 g) for 25 min. Then, PBMCs in the middle layer between the plasma and the separation fluid were collected and purified with red blood cell lysis buffer (TBD sciences, Tianjin, China). Cells were washed twice with 1X PBS, followed by centrifugation (5,000 rpm) for 10 min. PBMCs were cultured at 1 × 10^6^ cells/ml in complete RPMI 1640 culture medium (Gibco, Australia) supplemented with 10% fetal bovine serum (Gibco, Australia) and 1% penicillin/streptomycin. Cells were stimulated with 2 μl/ml (1 × 10^6^ cells/ml) leukocyte activation cocktail (550583; BD Biosciences, USA) and cultured at 37°C with 5% CO_2_ for 6 h, then harvested for flow cytometry analysis. Serum samples were obtained from all study participants and stored at −80°C until further analysis.

### Flow Cytometry

After 6 h incubation, PBMCs were stained with a marker of cell viability (Fixable Viability Stain 510 antibody) for 15 min at room temperature in the dark. Then, cells were stained for surface markers with APC-Cy7-anti-CD3 and BB515-anti-CD4 antibodies followed by fixation and permeabilization using the Cytofix/Cytoperm Soln Kit (BD Pharmingen) for 30 min at 4°C in the dark. After washing with permeabilization buffer, cells were stained for intracellular markers with PE-anti-IL-17A and APC-anti-IL-4 antibodies in permeabilization buffer for 30 min at 4°C in the dark. Isotype controls were employed in the control group. These antibodies were purchased from BD Pharmingen. Flow cytometry was performed, and data were analyzed using FACS Canto II (Becton Dickinson) and FlowJo version X software.

### Enzyme-Linked Immunosorbent Assay

The levels of MBD2, ECP and MPO in the serum of participants were determined by enzyme-linked immunosorbent assay (ELISA) using the Human MBD2 ELISA Kit (HUES03426; Genie, USA), Human ECP ELISA Kit (HUES02422; Genie, USA), Human MPO ELISA Kit (440007; Biolegend, USA). All experiments were performed according to the manufacturers' instructions.

### Statistical Analysis

SPSS 26.0 software (IBM Corp.) was used to perform all statistical analyses, and GraphPad Prism 8.0.1 software (GraphPad Software Inc) was used to generate the graphs. Continuous variables were described as the mean and standard deviation (SD) or median (interquartile range [IQR]), and categorical variables were expressed as the number (percentage). Differences between the three groups were determined by chi-square test, one-way analysis of variance (ANOVA) or Kruskal-Wallis test followed by Dunn's multiple comparisons test. Correlations between variables were analyzed by Spearman's rank correlation test. A *P*-value <0.05 indicated a statistically significant difference.

## Results

### Demographic Characteristics

Table 1 shows the demographic characteristics, lung function indexes and biochemical indexes of the 84 participants, including 21 HCs and 63 asthmatic patients (32 patients with asthma and 31 patients with severe asthma). The mean age was 50.76 ± 7.03 years for HCs, 58.78 ± 10.87 years for patients with asthma and 62.55 ± 9.85 years for patients with severe asthma (Table 1). There were significant differences in age, sex and BMI between the three groups. The IgE level was higher in patients with severe asthma and asthma compared to HCs, but FEV_1_, FEV_1_/FVC and FEV_1_%predicted were lower.

**Table 1:** Clinical characteristics of participants.

**Items**	**HCs (*n* = 21)**	**Asthma (*n* = 32)**	**Severe asthma (*n* = 31)**	***P* value**
Age(y), M ± SD	50.76 ± 7.03	58.78 ± 10.87	62.55 ± 9.85	<0.001
Sex M/F, n/n (%/%)	11/10(52.4/47.6)	13/19(40.6/59.4)	27/4(87.1/12.9)	<0.001
BMI (kg/m^2^), M ± SD	24.37 ± 2.87	24.87 ± 3.93	22.35 ± 3.27	0.013
**Smoking history, n (%)**				0.024
Never-smoker	14(66.7)	25(78.1)	14(45.2)	
Ex-smoker	7(33.3)	7(21.9)	17(54.8)	
ACT, M ± SD		22.68 ± 1.42	12.81 ± 3.13	<0.001
**Lung function indexes, median (IQR)**
FEV_1_ (L)	3.1(2.4–3.4)	1.7(1.5–2.0)	1.0(0.8–1.3)	<0.001
FEV_1_/FVC (%)	81.2(77.6–82.5)	69.0(57.8–73.8)	38.9(27.4–49.8)	<0.001
FEV_1_%predicted (%)	100.4(95.7–102.7)	73.7(65.7–85.2)	32.7(26.1–41.5)	<0.001
**Biochemical indexes, median (IQR)**
IgE (mg/l)	58.7(29.6–424.2)	114.3(63.7–804.9)	205.8(157.9–1275.0)	0.008
Blood eosinophils (× 10^9^)	0.15 ± 0.12	0.21 ± 0.19	0.36 ± 0.19	<0.001
Blood neutrophils (× 10^9^)	3.73 ± 0.83	4.36 ± 1.26	4.93 ± 1.70	0.01
Th2(%), M ± SD	1.02 ± 0.26	2.40 ± 0.27	3.69 ± 0.46	<0.001
Th17(%), M ± SD	0.99 ± 0.19	1.72 ± 0.41	3.26 ± 0.61	<0.001

*Comparisons were determined by chi-square test, one-way analysis of variance (ANOVA) or Kruskal-Wallis test followed by Dunn's multiple comparisons test. P <0.05 was considered statistically significant*.

*M, male; F, female; BMI, body mass index; ACT, asthma control test; FEV_1_, forced expiratory volume in 1 s; FVC, forced vital capacity; IgE, immune globulin E; Th2, T-helper cell type 2; Th17, T-helper cell type 17; M ± SD, mean ± standard deviation; IQR, interquartile range*.

Table 2 shows the demographic characteristics, lung function indexes and biochemical indexes of the 21 patients with T2 severe asthma and 10 patients with Th17 severe asthma. The mean age was 62.76 ± 9.34 years for patients with T2 severe asthma and 62.10 ± 11.36 years for patients with Th17 severe asthma (Table 2). There were no differences in age, sex or lung function between patients with T2 severe asthma and those with Th17 severe asthma. However, BMI, IgE, blood eosinophils and blood neutrophils were different between the two groups. Compared to patients with Th17 severe asthma, those with T2 severe asthma had a higher percentage of Th2 cells but a lower percentage of Th17 cells. However, patients with Th17 severe asthma had a higher percentage of Th17 cells but a lower percentage of Th2 cells compared to patients with T2 severe asthma. Detailed information regarding participant characteristics is shown in Tables 1, 2.

**Table 2:** Clinical characteristics of T2 and Th17 severe asthma participants.

**Items**	**T2 severe asthma (*n* = 21)**	**Th17 severe asthma (*n* = 10)**	***P* value**
Age(y), M ± SD	62.76 ± 9.34	62.10 ± 11.36	0.865
Sex M/F, n/n (%/%)	19/2(90.5/9.5)	8/2(80.0/20.0)	0.429
BMI (kg/m^2^), M ± SD	23.18 ± 3.38	20.61 ± 2.28	0.038
**Smoking history, n (%)**			0.690
Never-smoker	10(47.6)	4(40.0)	
Ex-smoker	11(52.4)	6(60.0)	
ACT, M ± SD	13.14 ± 3.48	12.10 ± 2.23	0.396
**Lung function indexes, median (IQR)**
FEV_1_ (L)	0.9(0.7–1.3)	1.0(0.9–1.2)	0.526
FEV_1_/FVC (%)	39.6(32.8–52.0)	33.4(24.7–39.5)	0.091
FEV_1_%predicted (%)	35.1(26.2–44.1)	30.8(24.5–35.9)	0.321
**Biochemical indexes, median (IQR)**
IgE (mg/l)	214.3(184.2–1290.4)	156.9(86.6–1184.3)	0.024
Blood eosinophils (× 10^9^)	0.41 ± 0.20	0.24 ± 0.10	0.015
Blood neutrophils (× 10^9^)	4.38 ± 1.55	6.09 ± 1.46	0.007
Th2 (%), M ± SD	3.86 ± 0.34	3.32 ± 0.47	0.001
Th17 (%), M ± SD	2.95 ± 0.42	3.92 ± 0.35	<0.001

*Comparisons were determined by chi-square test, one-way analysis of variance (ANOVA) or Kruskal-Wallis test followed by Dunn's multiple comparisons test. P <0.05 was considered statistically significant*.

*M, male; F, female; BMI, body mass index; ACT, asthma control test; FEV_1_, forced expiratory volume in 1 s; FVC, forced vital capacity; IgE, immune globulin E; T2, type 2; Th2, T-helper cell type 2; Th17, T-helper cell type 17; M ± SD, mean ± standard deviation; IQR, interquartile range*.

### Serum MBD2, ECP and MPO Levels in Different Groups

**Figure 2:**
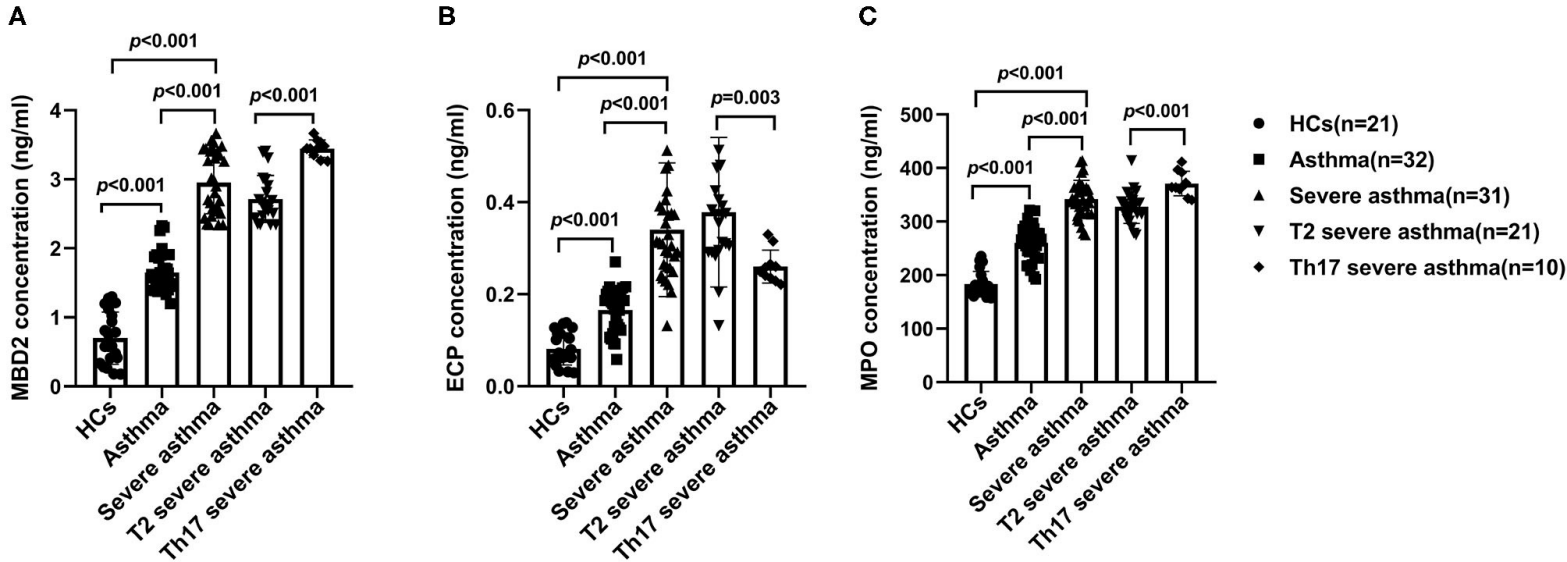
Serum MBD2, ECP and MPO levels in the different groups. MBD2 **(A)**, ECP **(B)** and MPO **(C)** levels were compared for HCs and patients with asthma, severe asthma, T2 severe asthma and Th17 severe asthma. Comparisons among the groups were performed by Kruskal-Wallis test followed by Dunn's multiple comparisons test. *P* < 0.05 was considered statistically significant. MBD2, methyl-CpG binding domain protein 2; ECP, eosinophilic cationic protein; MPO, myeloperoxidase.

Serum MBD2, ECP and MPO levels were increased in patients with severe asthma compared to those with asthma (*P* < 0.001) and HCs (*P* < 0.001), as well as in patients with asthma compared to HCs (*P* < 0.001; Figures 2A–C). Serum MBD2 and MPO levels were higher in patients with Th17 severe asthma compared to patients with T2 severe asthma (*P* < 0.001; Figures 2A,C), but serum ECP levels were lower in patients with Th17 severe asthma compared to those with T2 severe asthma (*P* = 0.003; Figure 2B).

### Percentage of Th2 and Th17 Cells in the Peripheral Blood of Different Groups

**Figure 3:**
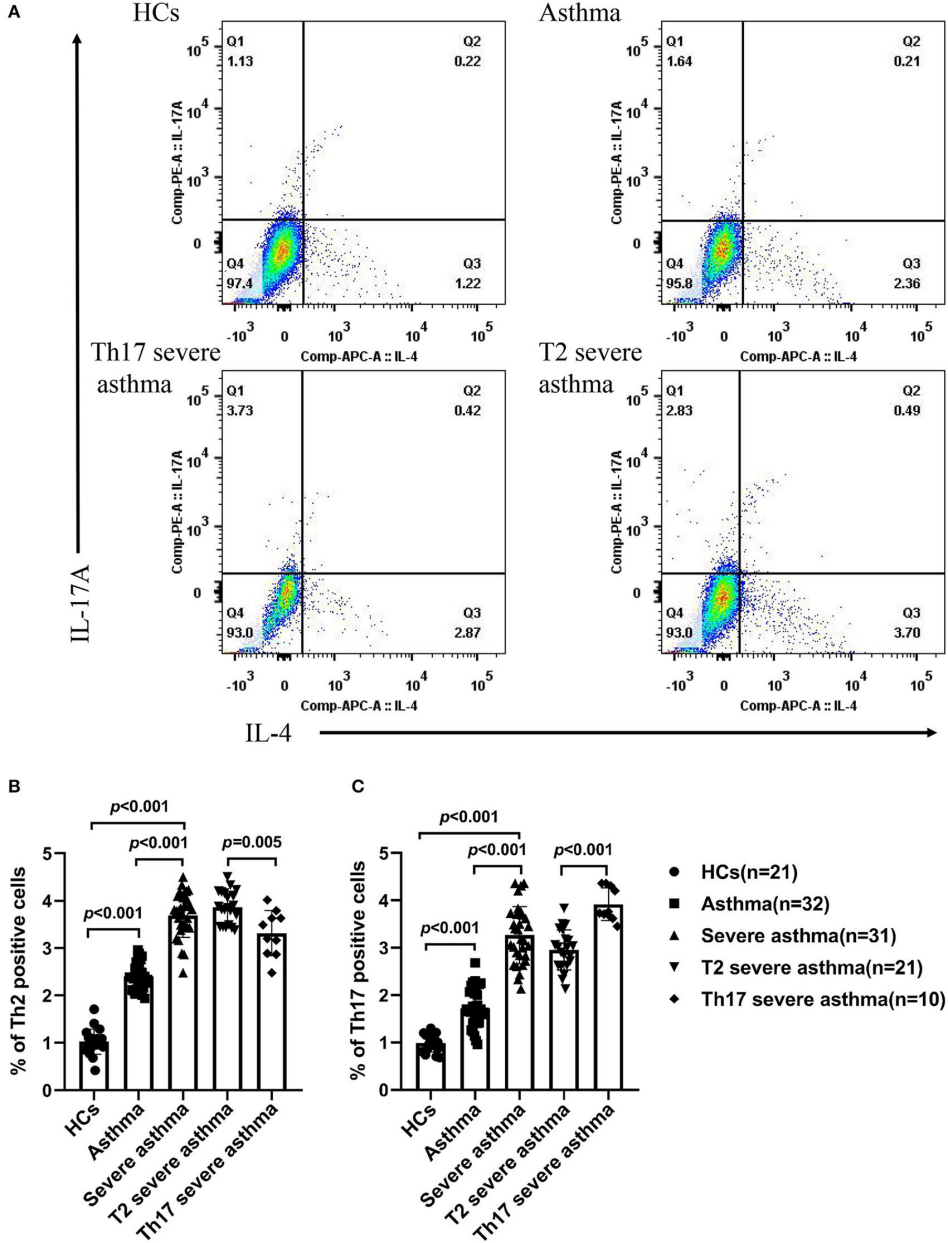
Percentage of Th2 and Th17 cells in the peripheral blood of different groups. **(A)** Flow cytometry used to measure the percentage of Th2 and Th17 cells in the peripheral blood of different groups. The percentage of Th2 cells **(B)** and the percentage of Th17 cells **(C)** were compared for HCs and patients with asthma, severe asthma, T2 severe asthma and Th17 severe asthma. Comparisons among the groups were performed by Kruskal-Wallis test followed by Dunn's multiple comparisons test. *P* < 0.05 was considered statistically significant. Th2, T-helper cell type 2; Th17, T-helper cell type 17.

The percentage of Th2 and Th17 cells in the peripheral blood was increased in patients with severe asthma compared to patients with asthma (*P* < 0.001) and HCs (*P* < 0.001), as well as in patients with asthma compared to HCs (*P* < 0.001; Figures 3A–C). In patients with asthma, the percentage of Th2 cells in the peripheral blood was higher than the percentage of Th17 cells (*P* < 0.001; Figures 3A–C). The percentage of Th17 cells in the peripheral blood was higher in patients with Th17 severe asthma than in patients with T2 severe asthma (*P* < 0.001) (Figures 3A,C), while the percentage of Th2 cells in the peripheral blood was lower in patients with Th17 severe asthma than in those with T2 severe asthma (*P* = 0.005; Figures 3A,B).

### Correlation of Serum MBD2 Levels With Clinical Characteristics and Pulmonary Function Test Results in Different Groups

**Figure 4:**
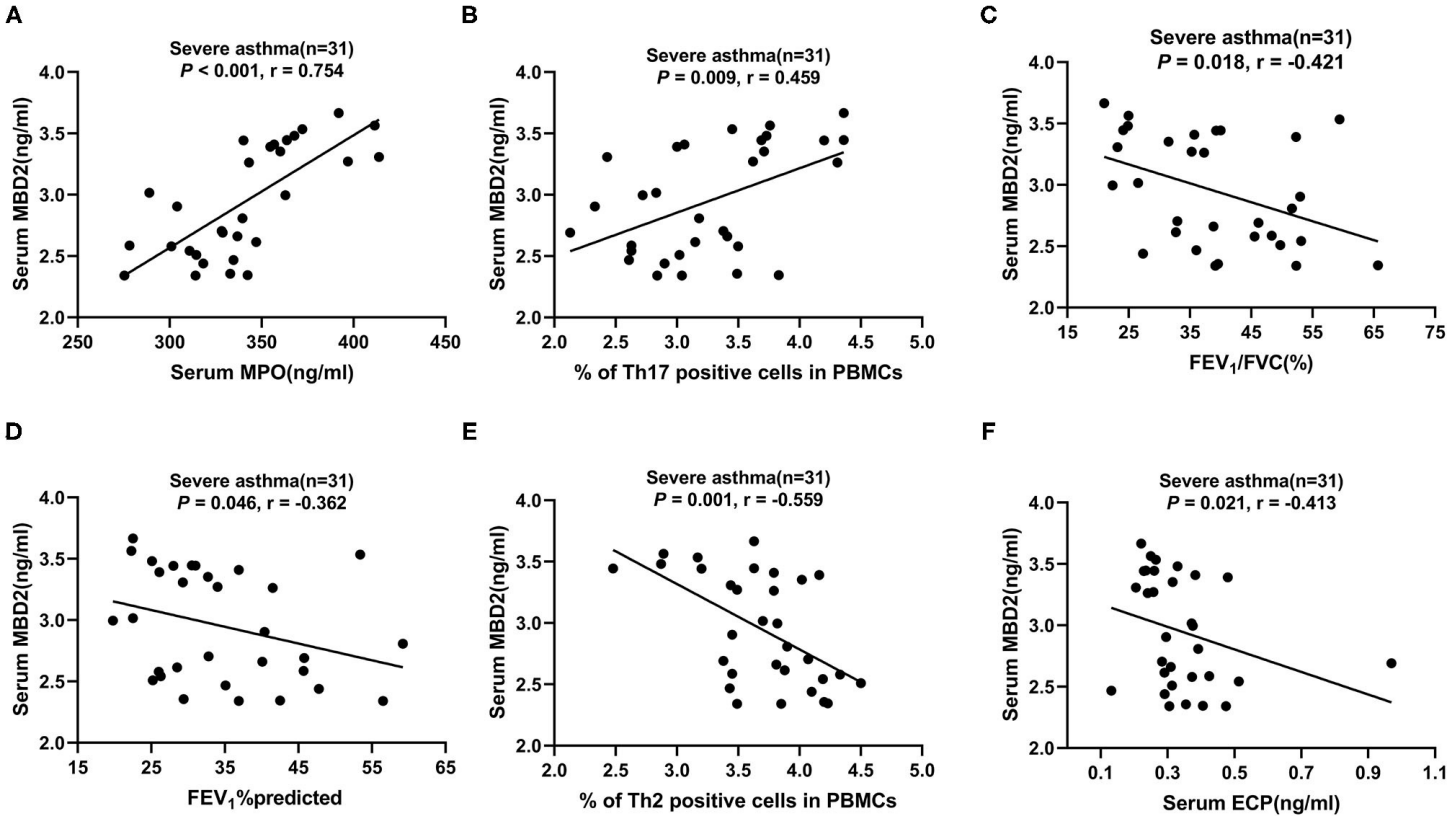
Correlation of serum MBD2 levels with clinical characteristics and pulmonary function test results in patients with severe asthma. **(A–F)** The correlation between serum MBD2 levels and serum MPO **(A)**, Th17 cells in PBMCs **(B)**, FEV_1_/FVC **(C)**, FEV_1_%predicted **(D)**, Th2 cells in PBMCs **(E)**, and serum ECP **(F)**. Correlations were determined by Spearman's rank correlation test. *P* < 0.05 was considered statistically significant. FEV_1_, forced expiratory volume in 1 s; FVC, forced vital capacity; MBD2, methyl-CpG binding domain protein 2; ECP, eosinophilic cationic protein; MPO, myeloperoxidase; Th2, T-helper cell type 2; Th17, T-helper cell type 17; r, correlation coefficient.

In patients with severe asthma, MBD2 levels were positively correlated with MPO (*r* = 0.750, *P* < 0.001; Figure 4A) and Th17 (*r* = 0.454, *P* = 0.010; Figure 4B), but negatively correlated with FEV_1_/FVC (*r* = −0.429, *P* = 0.016; Figure 4C), FEV_1_%predicted (*r* = −0.371, *P* = 0.040; Figure 4D), Th2 (*r* = −0.565, *P* = 0.001; Figure 4E) and ECP (*r* = −0.418, *P* < 0.019; Figure 4F; Table 3). There were no correlations between MBD2 levels and clinical parameters in HCs and patients with asthma. These data indicate that the serum MBD2 level is correlated with severe asthma.

**Table 3:** Correlation of serum MBD2 levels with clinical characteristics and pulmonary function test results in different groups.

	**MBD2 relative expression**					
	**HCs**		**Asthma**		**Severe asthma**	
**Items**	***P* value**	***r***	***P* value**	***r***	***P* value**	***r***
IgE	0.438	0.179	0.766	0.055	0.132	−0.277
FEV_1_/FVC	0.703	−0.088	0.306	−0.187	0.018	−0.421
FEV_1_%predicted	0.324	0.226	0.341	−0.174	0.046	−0.362
ECP	0.938	0.018	0.894	−0.025	0.021	−0.413
MPO	0.345	0.217	0.879	0.028	<0.001	0.754
Th2	0.607	−0.119	0.326	−0.179	0.001	−0.559
Th17	0.898	−0.030	0.639	−0.086	0.009	0.459

*Correlations were determined by Spearman's rank correlation test. P <0.05 was considered statistically significant*.

*IgE, immune globulin E; FEV_1_, forced expiratory volume in 1 s; FVC, forced vital capacity; MBD2, methyl-CpG binding domain protein 2; ECP, eosinophilic cationic protein; MPO, myeloperoxidase; Th2, T-helper cell type 2; Th17, T-helper cell type 17; r, correlation coefficient*.

## Discussion

Asthma is a highly heterogeneous disease characterized by chronic airway inflammation. Asthma is generally divided into T2 and non-T2 endotypes. The former is mainly mediated by Th2 cells and their secreted cytokines, interleukin (IL)-4, IL-5 and IL-13, and is mainly induced by the infiltration of eosinophils in the airway. This type of asthma is sensitive to glucocorticoid treatment and is known as eosinophilic asthma (25). On the other hand, the latter is caused by neutrophil infiltration rather than eosinophilic infiltration. This form of asthma is insensitive to glucocorticoid treatment and is mainly mediated by Th17 cells and their cytokine IL-17, also known as neutrophilic asthma. It often develops into severe asthma due to its lack of sensitivity to glucocorticoids (7, 8). In this study, we found that the percentage of Th2 and Th17 cells were increased in patients with severe asthma compared to those with asthma and HCs, as well as in asthma patients compared to HCs. The percentage of Th17 cells was higher in Th17 severe asthma compared to T2 severe asthma, but the percentage of Th2 cells was lower in Th17 severe asthma compared to T2 severe asthma. In this study, we also found that blood eosinophils and neutrophils were increased in patients with severe asthma compared to those with asthma and HCs, as well as in asthma patients compared to HCs. The count of blood neutrophils was higher in Th17 severe asthma compared to T2 severe asthma, but the count of blood eosinophils was lower in Th17 severe asthma compared to T2 severe asthma. These findings suggest that the presence of T2 and non-T2 endotypes in asthma, with the latter being primarily mediated by Th17 cells and neutrophil infiltration, which is consistent with previous studies (8, 26, 27). Th17 cells and their cytokines are involved in the steroid resistance mechanism of asthma through the induction of transforming growth factor-β expression to reduce apoptosis, while glucocorticoid therapy inhibits neutrophil apoptosis and enhances IL-17 production (28). In addition, phosphatidylinositol 4,5-bisphosphate 3-kinase (PI3K) enhances the production and secretion of IL-17A, which is activated by PI3K and leads to a decrease in histone deacetylase 2, thus rendering airway epithelial cells insensitive to glucocorticoids (29).

ECP and MPO are markers of eosinophil and neutrophil activity, respectively (14, 15). In this study, we found that serum ECP and MPO levels were increased in patients with severe asthma compared to those with asthma and HCs, as well as in asthma patients compared to HCs. Serum MPO levels were higher in Th17 severe asthma than in T2 severe asthma, but serum ECP levels were lower in Th17 severe asthma than in T2 severe asthma. These findings suggest that T2 asthma is accompanied by eosinophil activation while Th17 asthma is accompanied by neutrophil infiltration, which drives their respective inflammatory pathways. As a protein contained within eosinophil granules, ECP is strongly correlated with blood eosinophil counts (14). Studies have shown that the sputum ECP level is negatively correlated with asthma control. Patients with acute asthma had significantly higher levels of ECP and IL-5 than a control group, and serum ECP was found to be strongly dependent on IL-5. In addition, elevated blood eosinophil counts and ECP are associated with an increased rate of asthma exacerbations and increased symptoms (30–32). MPO is a heme-containing peroxidase, mainly expressed in neutrophils, which has been shown to be a local mediator of inflammation in tissue damage and a variety of resulting inflammatory diseases (33). Neutrophils can remove harmful pathogens by crowding out cytoplasm and nuclear material through a conserved cell death process distinct to necrosis and apoptosis, called neutrophil extracellular traps (NETs), and MPO is involved in the formation of NETs (34). Studies have shown a significantly greater increase in NETs and their components and chemokine ligand C-X-C motif chemokine ligand (CXCL)8/IL-8 in the induced sputum of patients with neutrophilic asthma compared to those with non-neutrophilic asthma, and this was negatively correlated with lung function (35). In addition, peripheral blood neutrophils from patients with severe asthma showed higher NETs than those from non-severe patients following *in vitro* CXCL8/ IL-8 stimulation, and NETs and neutrophil cytoplasts were positively correlated with IL-17 levels and biased toward Th17 differentiation (36, 37). Although MPO has not been extensively studied in asthma, these results suggest that MPO is likely to promote Th17 asthma.

As a “reader” and regulatory element of DNA methylation, MBD2 plays a regulatory role in T cell differentiation (38). In this study, we found that serum MBD2 levels were increased in patients with severe asthma compared to those with asthma and HCs, as well as in asthma patients compared to HCs. Serum MBD2 levels were higher in patients with Th17 severe asthma compared to those with T2 severe asthma. These findings suggest that MBD2 levels are significantly increased in severe asthma, especially in the Th17 endotype. We also confirmed that MBD2 levels are positively correlated with MPO and Th17 cells but negatively correlated with FEV_1_/ FVC, FEV_1_%predicted, Th2 cells and ECP in patients with severe asthma. MPO is a marker of neutrophil activity, and the correlation analysis suggested that MBD2 might be involved in the development of asthma by mediating the differentiation of Th17 cells, which secrete the cytokine IL-17A to chemotactic neutrophils into the airway and causing chronic inflammation. In addition, MBD2 was found to be associated with poor lung function in patients with severe asthma. MBD2 is highly conserved and widely expressed, and it plays a key role in inflammation by interacting with nucleosome remodeling and histone deacetylase complexes as inhibitors that lead to transcriptional silencing (39). Systemic lupus erythematosus (SLE) is an autoimmune disease, and a study by Qin et al. found that elevated MBD2 mRNA levels in the CD4^+^ T cells of SLE patients were negatively correlated with global DNA methylation and positively correlated with the SLE disease activity index (40). In addition, the proportion of regulatory T cells (Treg) in the spleen was significantly reduced after MBD2 knockout (41). Surprisingly, MBD2-deficient mice did not develop autoimmunity, possibly because their T effector cells were insensitive to stimulation and susceptible to Treg suppression. Inflammation that causes the mobilization and recruitment of immune cells is one of the main features of chronic obstructive pulmonary disease (COPD). Zeng et al. found that the expression of MBD2 was reduced in the airway epithelium of patients with COPD, while in human bronchial epithelial cells, this reduced expression was related to increases in IL-6 and IL-8 mediated by the extracellular signal-regulated kinase and p38 mitogen-activated protein kinase pathways (42). Wen et al. found that MBD2 levels were decreased in patients with acute exacerbation of COPD, and MBD2 was positively correlated with miR-301a-5p and negatively correlated with CXCL12. Overexpression of MBD2 significantly promoted the production of miR-301a-5p in human bronchial epithelial cells, but inhibited the production of CXCL12. CXCL12 was confirmed to be a direct target of miR-301a-5p (43). These results indicate that MBD2 may contribute to chronic airway inflammation in COPD.

To our knowledge, this study is the first to report an association between serum MBD2 expression and patients with Th17 severe asthma. Studies have shown that MBD2 is significantly increased in neutrophilic severe asthma mouse models, it is positively correlated with IL-17A and it can regulate Th17 cell differentiation through its downstream molecules, such as transcription factor hypoxia-inducible factor 1α and suppressor of cytokine signaling 3 (13, 44). Th17 severe asthma is often characterized by poor response to treatment and impaired lung function due to insensitivity to glucocorticoids, which seriously affects the quality of life of asthmatic patients. However, there is still a lack of convenient and reliable biomarkers for the rapid identification of Th17 severe asthma. The induced sputum cell count can be used to screen for neutrophilic asthma endotypes; however, not all asthmatic patients are suitable for this test or tolerant to induced sputum (45). In this study, we confirmed that MBD2 is expressed in the serum of patients with asthma, suggesting that MBD2 is involved in the development of asthma. In addition, we found that MBD2 was significantly increased in severe asthma, especially the Th17 endotype, compared to HCs and patients with mild to moderate asthma. Moreover, MBD2 was positively correlated with Th17 cells and MPO, which is of great significance for the clinical diagnosis and treatment of Th17 severe asthma.

In this study, we found that serum MBD2, ECP and MPO levels and the percentage of Th2 cells in the peripheral blood was higher in patients with asthma and in those with T2 severe asthma. These findings suggest that the asthma cases were mainly allergic, and that both Th17 cells and neutrophils might be involved in Th2 cell-mediated eosinophilic infiltration in allergic asthma. Renata et al. found that MPO was decreased in the ovalbumin group compared to the control group in an experimental allergic asthma model (46). However, the role of MPO in allergic asthma is yet to be studied. Interestingly, in this study, we also found that MBD2 was negatively correlated with Th2 cells and ECP in patients with severe asthma. This may have occurred through an effect of MBD2 on the activity of eosinophils and even the function of Th2 cells through downstream target genes while promoting the differentiation of Th17 cells. Cook et al. found that in the absence of MBD2 expression, dendritic cells (DCs) showed reduced phenotypic activation and significantly impaired ability to activate Th2 against worms or allergens (38). These results suggest that MBD2 indirectly affects the maturation of CD4^+^ T cells by regulating gene expression in DCs, including its role in controlling the ability of DCs to induce the Th2 response. In another study, Peter et al. found that the injection of anti-IL-17 monoclonal antibodies prior to allergen inhalation significantly reduced bronchial neutrophilic induction, but unexpectedly increased IL-5 production, exacerbating allergen-induced bronchial eosinophilic inflammation (47). The mechanism of MBD2 regulation of Th2 cell function and eosinophilic inflammation in asthma needs to be further elucidated.

There were some limitations in this study: (a) This study was a single-center case-control study with patients from one region; therefore, the results of the study might not be representative of the whole country. (b) Patients with acute attack and cardiac asthma were excluded from our study; thus, the results may not be applicable to all asthma patients. (c) Airway inflammation was not assessed in this study, and the correlation between MBD2 and inflammation in asthmatic patients could be further evaluated by measuring fractionated exhaled nitric oxide.

## Conclusions

In conclusion, serum MBD2 may be a potential new biomarker for identifying severe asthma, Th17 severe asthma and the type of airway inflammation. However, these findings are still preliminary and need to be further investigated.

## Data Availability Statement

The original contributions presented in the study are included in the article/supplementary material, further inquiries can be directed to the corresponding author/s.

## Author Contributions

ZC, SL, XZ, and XX: conceptualization and design. ZC, BX, LM, QZ, SL, XZ, and XX: methodology. ZC, YY, YH, JL, DL, YL, SL, XZ, and XX: data management. ZC, BW, WD, JJ, DZ, SL, XZ, and XX: statistical analysis and interpretation. All authors contributed to drafting the original manuscript of important intellectual content and final approval of the manuscript.

## Funding

This study was supported by the National Natural Science Foundation of China (Nos. 81960006 and 81760009) and the Fundamental Research Funds for the Central Universities of Central South University (No. 2020zzts881).

## Conflict of Interest

The authors declare that the research was conducted in the absence of any commercial or financial relationships that could be construed as a potential conflict of interest.

## Publisher's Note

All claims expressed in this article are solely those of the authors and do not necessarily represent those of their affiliated organizations, or those of the publisher, the editors and the reviewers. Any product that may be evaluated in this article, or claim that may be made by its manufacturer, is not guaranteed or endorsed by the publisher.

## Abbreviations

ACT: asthma control test
BMI: body mass index
COPD: chronic obstructive pulmonary disease
CXCL8: chemokine ligand C-X-C motif chemokine ligand8
DCs: dendritic cells
ECP: eosinophilic cationic protein
ELISA: enzyme-linked immuno-sorbent assay
FEV1: forced expiratory volume in 1 second
FVC: forced vital capacity
IgE: immune globulin E
IL-4: interleukin-4
IQR: interquartile range
MBD2: methyl-CpG binding domain protein 2
MPO: myeloperoxidase
NETs: neutrophil extracellular traps
PBMCs: peripheral blood mononuclear cells
PFT: pulmonary function test
PI3K, phosphatidylinositol 4: 5-bisphosphate 3-kinase
SLE: systemic lupus erythematosus
T2: type 2
Th17: T-helper cell type 17
Th2: T-helper cell type 2
Treg: regulatory T cells
PBS: phosphate-buffered saline.
